# Genome sequences of *Rhizopogon roseolus*, *Mariannaea elegans, Myrothecium verrucaria*, and *Sphaerostilbella broomeana* and the identification of biosynthetic gene clusters for fungal peptide natural products

**DOI:** 10.1093/g3journal/jkac095

**Published:** 2022-04-26

**Authors:** Eva Vogt, Christopher M Field, Lukas Sonderegger, Markus Künzler

**Affiliations:** Institute of Microbiology, Department of Biology, ETH Zürich, Zürich CH-8093, Switzerland; Institute of Microbiology, Department of Biology, ETH Zürich, Zürich CH-8093, Switzerland; Institute of Microbiology, Department of Biology, ETH Zürich, Zürich CH-8093, Switzerland; Institute of Microbiology, Department of Biology, ETH Zürich, Zürich CH-8093, Switzerland

**Keywords:** ribosomally synthesized and posttranslationally modified peptide, nonribosomal peptide, peptaibols, verrucamides, mariannamides, broomeanamides, *Rhizopogon roseolus*, *Mariannaea elegans*, *Myrothecium verrucaria*, *Sphaerostilbella broomeana*

## Abstract

In recent years, a variety of fungal cyclic peptides with interesting bioactivities have been discovered. For many of these peptides, the biosynthetic pathways are unknown and their elucidation often holds surprises. The cyclic and backbone *N*-methylated omphalotins from *Omphalotus olearius* were recently shown to constitute a novel class (borosins) of ribosomally synthesized and posttranslationally modified peptides, members of which are produced by many fungi, including species of the genus Rhizopogon. Other recently discovered fungal peptide macrocycles include the mariannamides from *Mariannaea elegans* and the backbone *N*-methylated verrucamides and broomeanamides from *Myrothecium verrucaria* and *Sphaerostilbella broomeana*, respectively*.* Here, we present draft genome sequences of four fungal species *Rhizopogon roseolus*, *Mariannaea elegans, Myrothecium verrucaria*, and *Sphaerostilbella broomeana.* We screened these genomes for precursor proteins or gene clusters involved in the mariannamide, verrucamide, and broomeanamide biosynthesis including a general screen for borosin-producing precursor proteins. While our genomic screen for potential ribosomally synthesized and posttranslationally modified peptide precursor proteins of mariannamides, verrucamides, broomeanamides, and borosins remained unsuccessful, antiSMASH predicted nonribosomal peptide synthase gene clusters that may be responsible for the biosynthesis of mariannamides, verrucamides, and broomeanamides*.* In *M. verrucaria*, our antiSMASH search led to a putative NRPS gene cluster with a predicted peptide product of 20 amino acids, including multiple nonproteinogenic isovalines. This cluster likely encodes a member of the peptaibols, an antimicrobial class of peptides previously isolated primarily from the Genus *Trichoderma*. The nonribosomal peptide synthase gene clusters discovered in our screenings are promising candidates for future research.

## Introduction 

Borosins, a class of backbone *N*-methylated ribosomally synthesized and posttranslationally modified peptides (RiPPs), were defined in 2017 following the discovery of the biosynthesis pathway of the founding member omphalotin A (Ramm *et al.* 2017; Van Der Velden *et al.* 2017). This nematotoxic peptide macrocycle and its variants are produced by the fungus *Omphalotus olearius* via the self-modifying precursor protein OphMA. OphMA contains an N-terminal αN-methyltransferase domain that methylates the precursor’s C-terminal core peptide, followed by cleavage, cyclization and release of omphalotin (Van Der Velden *et al.* 2017). Backbone *N*-methylations were previously found exclusively in nonribosomal peptides and were even considered a hallmark of this type of peptides. Therefore, it was a surprise to find them in RiPPs (Vogt and Künzler 2019). Genome mining led to the discovery of many other potential OphMA-like peptide precursors in fungi, including *Dendrothele bispora* and *Lentinula edodes* (Quijano *et al.* 2019). The genomes of these fungi contain biosynthetic gene clusters with similar composition and organization as the omphalotin cluster. In addition, the encoded OphMA homologs contain core peptide with high sequence similarity to omphalotin A. Analysis of fungal tissue samples confirmed the production of the corresponding peptides, termed dendrothelins and lentinulins (Matabaro *et al.* 2021). Recent publications demonstrated the presence of borosin clusters with trans-acting αN-methyltransferases in bacteria (Cho *et al.* 2022; Imani *et al.* 2022). Based on these findings, we were interested in investigating previously discovered, backbone *N*-methylated cyclic peptides that were hypothesized to be of nonribosomal origin, to represent novel members of the borosin class of RiPPs.

**Fig. 1.**
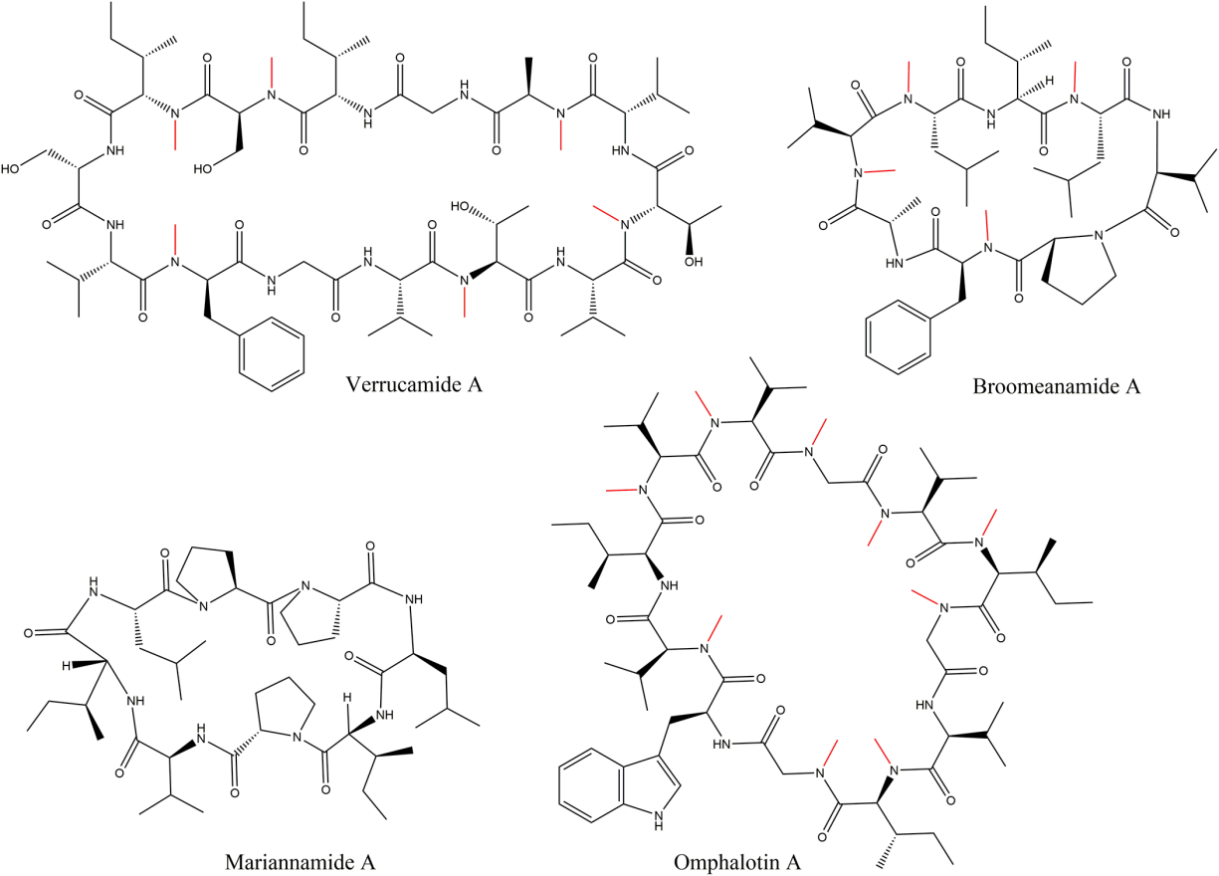
Structures of the cyclic fungal peptides verrucamide A (Zou *et al.* 2011), broomeanamide A (Ekanayake *et al.* 2021), mariannamide A (Ishiuchi *et al.* 2020), and omphalotin A (Van Der Velden *et al.* 2017), the founding member of the borosin class of RiPPs. Backbone *N*-methylations are indicated in red.

Recently discovered cyclic, backbone *N*-methylated peptides include verrucamides A-D, tetradecapeptides that are produced by the ascomycete *Myrothecium verrucaria* and contain two D-configured amino acids (Zou *et al.* 2011), and the octapeptides broomeanamides A-C from the mycoparasitic ascomycete *Sphaerostilbella broomeana* where all eight amino acids are L-configured (Fig. 1) (Ekanayake *et al.* 2021). Another class of cyclic peptides are the octapeptides mariannamides A and B isolated from the filamentous ascomycete *Mariannaea elegans* that are also composed of all L-amino acids amongst three proline residues but do not contain any backbone *N*-methylations (Fig. 1) (Ishiuchi *et al.* 2020)*.* Both verrucamides and mariannamides were shown to possess antibacterial properties (Zou *et al.* 2011; Ishiuchi *et al.* 2020). The mode of synthesis of all three peptide classes is unknown; the structural similarity of the verrucamides and broomeanamides to the cyclic, backbone *N*-methylated borosins indicated that they may be RiPPs, although the presence of D-amino acids in the verrucamides rather suggested a nonribosomal origin. Only one fungal RiPP class with a residue in D-configuration has been identified so far (phallotoxins, Hallen *et al.* 2007).

Here, we report the genome sequences of *M. verrucaria*, *M. elegans*, *Rhizopogonroseolus*, and *S. broomeana*. We mined the genomes of *M. verrucaria*, *M. elegans*, and *S. broomeana* for potential RiPP precursor proteins of the verrucamides, mariannamides, and broomeanamides, respectively. In addition, we performed an antiSMASH search to screen for nonribosomal peptide (NRP) biosynthetic gene clusters that might encode genes for verrucamide, mariannamide, and broomeanamide synthesis. We sequenced the genome of the agaricomycete *R.roseolus*, as the genomes of two species of the genus *Rhizopogon* were shown in BLAST searches to encode multiple OphMA homologs each (Quijano *et al.* 2019). Finally, we performed screens to find new OphMA homologs in *R. roseolus, M. verrucaria*, *M. elegans*, and *S. broomeana*.

## Materials and methods

### Strains and cultivation

The sequenced strains of *M. verrucaria*, *M. elegans*, and *S. broomeana* are the authentic producers of the verrucamides, mariannamides, and broomeanamides as seen in Zou *et al.* (2011), Ishiuchi *et al.* (2020), and Ekanayake *et al.* (2021)*. Myrothecium verrucaria* DSM 2087 was received by the Leibniz Institute DSMZ-German Collection of Microorganisms and Cell Cultures, Germany, *M.elegans* NBRC102301 was ordered from the Biological Resource Center, National Institute of Technology and Evaluation (NITE), Japan, heterokaryotic *R.roseolus* Mykothek Nr 97.03 (CBS 149159) was received from Martina Peter from the Eidgenössische Forschungsanstalt für Wald, Schnee und Landschaft (WSL), Switzerland. *Sphaerostilbella broomeana* TFC201724 is deposited at the Tartu Fungal Culture Collection (TFC) of the University of Tartu, Estonia, and was received by Kadri Põldmaa from the University of Tartu. *Myrotheciumverrucaria* was cultivated on Corn Meal Agar (CMA) at 30°C, *M. elegans* NBRC102301 on Potato Dextrose Agar (PDA) at room temperature, *R. roseolus* on Yeast Malt Agar (YMG) at room temperature, and *S. broomeana* on PDA at room temperature.

### Sample preparation and sequencing

The fungi were cultivated on cellophane-covered agar plates before their mycelia were harvested. *Myrotheciumverrucaria*, *M. elegans*, and *R. roseolus* mycelia were harvested after 14, 9, and 40 days, respectively, and lysed by grinding with a mortar and pestle in the presence of liquid nitrogen. *S. broomeana* mycelium was harvested after 7 days, mixed in an Eppendorf tube with 0. 5 mm glass beads, frozen in liquid nitrogen and then lysed by vigorous shaking in a Fastprep machine for 2 times 45 s at level 6. Genomic DNA was extracted using the QIAGEN DNeasy plant Mini kit, DNA concentration measured using a Qubit dsDNA kit and DNA quality confirmed by running a fraction of the DNA on an agarose gel. The DNA was sent to Novogene, United Kingdom, for shotgun sequencing on an Illumina Novaseq, producing paired-end 150 bp reads, aiming for approximately 100x coverage.

### Quality control

BBDuk (v38.87, Joint Genome Institute) was first used in right-trimming mode with a kmer length of 23 down to 11 and a hamming distance of 1 to filter out sequencing adapters. A second pass with a kmer length of 31 and a hamming distance of 1 was used to filter out PhiX sequences. A third and final pass performed quality trimming on both read ends with a Phred score cutoff of 14 and an average quality score cutoff of 20, with reads under 45 bp or containing Ns subsequently rejected.

### Assembly

The paired-end and singleton reads of each read set were assembled using SPAdes (v3.14.0) (Nurk *et al.* 2013) in isolate mode, but otherwise default parameters.

### Gene calling

GlimmerHMM (v3.0.4) (Majoros *et al.* 2004) was trained on exon sequences taken from phylogenetically close reference genomes (*Mariannaea sp.* strain PMI_226 v1.0 for *M. elegans*; *Myrothecium inundatum* CBS 120646 v1.0 for *M. verrucaria*; *Rhizopogon vulgaris* FC72 v1.0 for *R. roseolus*; *Trichoderma reesei* QM6a NW_006711148.1 for *S. broomeana*) and subsequently used to call genes in the genome assemblies. All reference genomes were obtained from JGI (Nordberg *et al.* 2014) in August 2020 (*M. elegans, M. verrucaria*, and *R. roseolus*) and October 2021 (*S. broomeana*). Average nucleotide identity (ANI) between assemblies and reference genomes were performed with FastANI (Jain *et al.* 2018) that implements a similar calculation to Goris *et al.* (2007).

### Quality assessment

Completeness of the genome assemblies was assessed using BUSCO (v5.0.0) (Simão *et al.* 2015) in genome mode with the –auto-lineage-euk parameter to automatically assess the likely lineage of each strain (*M. elegans*, *M. verrucaria*, *S. broomeana*: hypocreales; *R. roseolus*: boletales). To test for bacterial contamination, the tool mOTUs (v3.0.0) (Milanese *et al.* 2019) was run on the reads for each sample. *M. elegans* had 2 inserts that could not be assigned to a specific mOTU; *M. verrucaria* had 1 insert corresponding to “*Phyllobacterium species incertae sedis*”; *R. roseolus* and *S. broomeana* returned no hits. These very low hit counts indicate that it is very unlikely for there to be any contamination by bacteria in the samples.

### Taxonomic analysis

The *ssu_finder* function of CheckM (v1.0.13) (Parks *et al.* 2015) was used to extract 16S and 18S rRNA gene sequences from the assemblies. 18S sequences of 1,726, 1,725, and 1,726 bp were found for *M. elegans*, *M. verrucaria*, and *S.broomeana*, respectively, but no such sequence was found for *R. roseolus*, likely because its assembly was highly fragmented. The sequences were aligned with the SILVA taxonomy database (v138) (Quast *et al.* 2013) using the provided software SINA (v1.6.1) (Pruesse *et al.* 2012). The internal transcribed spacer (ITS) region of each strain was extracted from its assembly using ITSx (Bengtsson-Palme *et al.* 2013) and its Fungi profile set. The sequences were then analyzed with the UNITE database (Nilsson *et al.* 2019; Kõljalg *et al.* 2020).

### Genome mining

To search for all possible arrangements of cyclic peptides of interest, a custom Python script generated a fasta file containing all possible variants of linearized peptide sequences for the verrucamides, mariannamides, and broomeanamides. For broomeanamide A, for example, these sequences would be VPFAVLIL, PFAVLILV, FAVLILVP, and so on. First, the predicted protein sequences were searched for all peptides with *blastp*, then the assemblies were searched for all peptides with *tblastn*, both part of the BLAST+ suite (v2.11.0) (Camacho *et al.* 2009). As a positive control for the functionality of our mining method, we screened the genome of *O.olearius* using the peptide sequence of the cyclic RiPP omphalotin (Ramm *et al.* 2017; Van Der Velden *et al.* 2017). We found the omphalotin precursor protein OphMA, thus confirming that our method works. The 300 residue long N-terminal methyltransferase domain of the protein OphMA from *O.olearius* (Quijano *et al.* 2019) was searched for in the predicted protein sequences with *blastp*. Further, all assemblies were analyzed with the fungal version of antiSMASH (v5.1.0) (Blin *et al.* 2019), which ignores contigs of less than 1 kbp in length by default, to look for biosynthetic gene clusters.

## Results and discussion

### Genome assembly and completeness

The genomes of the fungi *M.verrucaria*, *M.elegans*, *R.roseolus*, and *S.broomeana* were sequenced using Illumina PE150 and assembled using the reference genomes *M.inundatum* CBS 120646 v1.0, *Mariannaea sp.* strain PMI_226 v1.0, Rh*izopogon vulgaris* FC72 v1.0 and *T.reesei* QM6a. The *M. verrucaria* assembly had the highest quality of the 4 assemblies with the lowest contig count of 3,197 and the highest N50 value of 1,066,851, while *M. elegans*, *R. roseolus*, and *S. broomeana* had contig counts of 3,718, 23,000, and 3,882, respectively, and N50 values of 288,335, 61,999, and 295,130 (Table 1). As an alternative assessment of genome assembly and annotation completeness, the open-source software Benchmarking Universal Single-Copy Orthologs (BUSCO) was used (Simão *et al.* 2015). *Myrotheciumverrucaria* was 98.0% complete as a Eukaryote, while *M. elegans*, *R. roseolus*, and *S. broomeana* were 99.2%, 95.7%, and 98.0% complete, respectively. Completeness for the order Hypocreales (for *M. verrucaria*, *M.elegans*, and *S. broomeana*) and Boletales (for *R. roesolus*) was 96.6%, 97.4%, 97.5%, and 95.1%, respectively. Potential 16S rRNA sequences were extracted from the assembly to confirm the absence of bacterial contamination.

**Table 1.** Summary of the assembled genomes of the 4 newly sequenced fungal species *M. verrucaria*, *M. elegans, R. roseolus*, and *S. broomeana*.

		*M. verrucaria*	*M. elegans*	*R. roseolus*	*S. broomeana*
All scaffolds	Count	3,197	3,718	23,000	3,882
	Length	46,297,313	52,632,238	37,675,430	36,266 ,877
	N50	1,066,851	288,335	61,999	295,130
	N90	2,846,945	1,147,592	169,220	800,567
	Max	4,080,732	2,068,507	547,319	1,217,981
Scaffolds ≥ 1 kbp	Count	434	1,098	1,543	1,121
	Length	45,599,860	51,857,879	33,959,226	35,139,140
	N50	1,066,851	294,018	70,907	311,573
	N90	2,846,945	1,147,592	173,881	800,567
	Max	4,080,732	2,068,507	547,319	1,217,981
BUSCO	Completeness	98.0% (Eukaryotes)	99.2% (Eukaryotes)	95.7% (Eukaryotes)	98.0% (Eukaryotes)
	Completeness	96.6% (Hypocreales)	97.4% (Hypocreales)	95.1% (Boletales)	97.5% (Hypocreales)
	Single copy	95.5%	96.5%	93.8%	97.3%
	Duplicated	1.1%	0.9%	1.3%	0.2%
	Fragmented	0.4%	0.3%	0.6%	0.2%
	Missing	3.0%	2.3%	4.3%	2.3%
	Number of searched genes	4,494	4,494	4,878	4,494
Average nucleotide identity (ANI)		78.8% (*M. inundatum)*	79.9% (*Mariannaea* sp.)	90.2% (*Rhizopogon vulgaris)*	<70% (*T. reesei)*

Separate values are given for all scaffolds and scaffolds with a size of 1 kbp or more. Given parameters are the scaffold count, scaffold length, N50 and N90 values, and maximum scaffold length. The N50 and N90 values describe assembly contiguity by giving the minimal contig size that, together with all larger contigs, covers 50% or 90% of the total genome, respectively. BUSCO values describe the assembly completeness compared to Eukaryotes or the orders Hypocreales or Boletales. Average nucleotide identity describes nucleotide similarity to the reference genomes.

*Myrothecium* *verrucaria*, *M. elegans*, and *R. roseolus* had an average nucleotide identity (ANI) of 78.8%, 79.9%, and 90.2% with their reference fungi *M.inundatum*, *Mariannaea sp. strain PMI_226* and *Rhizopogon vulgaris* (Table 1)*.* No ANI value could be generated for *S. broomeana* and its reference strain *T.reesei*, meaning that sequence similarity between the 2 strains is too low (<70%). For a deeper taxonomical analysis of the sequenced species, the 18S rRNA sequences were extracted and ran against the SILVA ribosomal RNA taxonomy database (Quast *et al.* 2013). *Myrotheciumverrucaria* was identified as a member of the genus Myrothecium, *M. elegans* as a member of the order Hypocreales, *R. roseolus* as a member of either the order Boletales or Agaricales and *S. broomeana* as a member of the genus Trichoderma.

### Screenings for RiPP precursors and NRP biosynthetic gene clusters

All 4 genomes were screened for potential RiPP precursor proteins; *M. verrucaria* for verrucamide precursors, *M. elegans* for mariannamide precursors, *S. broomeana* for broomeanamide precursors, and all 4 genomes, including *R. roseolus*, for OphMA homologs. Screens were performed using all circular permutations of verrucamide, mariannamide, and broomeanamide sequences. In addition, all genomes were screened for the N-terminal methyltransferase domain of OphMA. These searches yielded no hits, indicating that the cyclic backbone *N*-methylated verrucamides and broomeanamides and the cyclic mariannamides are not genetically encoded and therefore may indeed be NRPs, and that *R. roseolus*, unlike many of its relatives from the genus Rhizopogon, does not contain any OphMA homologs.

Following the unsuccessful search for RiPP precursors of verrucamides, mariannamides, and broomeanamides, an additional search was performed using the fungal version of the “antibiotics and secondary metabolite analysis shell” antiSMASH (Blin *et al.* 2019) with the goal of finding NRP biosynthetic gene clusters that might direct the biosynthesis of the isolated peptide natural products. antiSMASH currently uses an ensemble prediction method integrating several algorithms to predict the substrate specificity of adenylation domains (Blin *et al.* 2017). In *M. verrucaria*, 1 NRP cluster was predicted to produce a verrucamide-like peptide with the correct length and several *N*-methylated residues, whereas in *M. elegans*, 1 cluster was predicted to produce a peptide of the same length as the mariannamides, containing several leucines and at least 1 proline (Table 2). In *S. broomeana*, cluster NRPS 77.1 was predicted to produce a 6-residue-long peptide containing 1 isoleucine, 1 leucine and a total of 4 *N*-methlyations. The broomeanamides are longer (8 residues), but contain 4 *N*-methylated residues, 2 leucines and, in the case of Broomeanamide A, 1 isoleucine (Table 2).

**Table 2.** *Myrothecium verrucaria, M. elegans*, and *S. broomeana* possess candidate NRP biosynthetic gene clusters for the synthesis of verrucamide-like, mariannamide-like, and broomeanamide-like peptides.

*M. verrucaria* NRPS 2.4	
Hit region	NODE_2_length_2846945_cov_41.963601—Region 4—NRPS
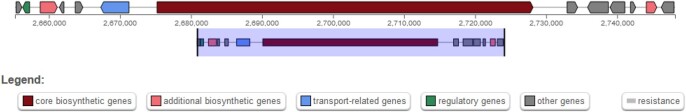	
NRP predicted seq.	A? ? V ? I ? ?? ? ??I F
Verrucamide A	A GI SI SV F GV T V T V
Verrucamide B	A GV SI SV F GV T V T V
Verrucamide C	A GI SV SV F GV T V T V
Verrucamide D	A GV SV SV F GV T V T V

*M. elegans* NRPS 180.1	
Hit region	NODE_180_length_54293_cov_43.947949—Region 1—NRPS
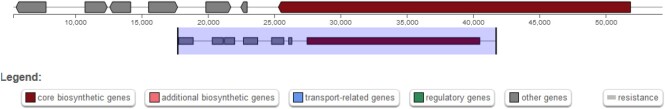	
NRP predicted seq.	LL??LP?L
Mariannamide A	IPVILPPL
Mariannamide B	IPVVLPPL

*S. broomeana* NRPS 77.1	
Hit region	NODE_77_length_123022_cov_43.474692.gene39—Region 1—NRPS
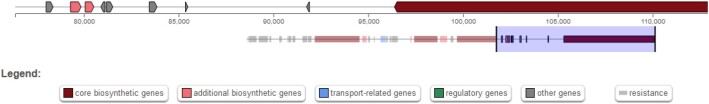	
NRP predicted seq.	? ? ?I ? L
Broomeanamide A	V P F AV L I L
Broomeanamide B	V P F AV L V L

A comparison between the predicted product sequence and the known sequences of verrucamide A-D, mariannamide A-B and broomeanamide A-B is given (Zou *et al.* 2011; Ishiuchi *et al.* 2020). Red letters indicate *N*-methylated residues, underlined letters represent D-amino acids. Question marks indicate non-specified residues.

Another NRP biosynthetic gene cluster of *M. verrucaria* was predicted to encode a 20 residue peptide with 11 residues of the nonproteinogenic amino acid isovaline (Table 3). This peptide is likely a peptaibol. Peptaibols are a class of antimicrobial NRPs from fungi that are 5–20 residues long, linear, N- and C-terminally modified with amino alcohol groups, and defined by the presence of the non‐proteinogenic amino acids α‐aminoisobutyric acid (Y) and/or isovaline (X) (de la Fuente-Núñez *et al.* 2013). The predicted peptide from *M. verrucaria* does not contain α‐aminoisobutyric acid, but 11 residues of isovaline. There are no known peptaibols with such a high content of isovaline, so it is likely that some of these predicted isovalines are rather alpha-aminoisobutryric acids or other residues. To date, over 1,000 peptaibols have been characterized in various members of the order Hypocreales, with the vast majority produced by members of the genus Trichoderma (de la Fuente-Núñez *et al.* 2013). Our antiSMASH search suggested 2 additional isovaline-containing NRPs in *M.verrucaria* (NRPS 3.4, X? X? L? Q? X and NRPS 15.2, XQ? X???) and 1 in *S.broomeana* (NRPS 31.1, XXX? X? QX??). Peptaibols have been previously reported in *Sphaerostilbella toxica* (Perlatti *et al.* 2020), but to our knowledge no peptaibols have been described in the Genus Myrothecium.

**Table 3.** Predicted NRP biosynthetic gene cluster in *M. verrucaria* encoding a putative peptide of the peptaibol class, containing the nonproteinogenic amino acid isovaline (X).

*M. verrucaria* NRPS 13.3	
Hit region	NODE_13_length_1066851_cov_42.619387- Region 3 - NRPS. Location: 807,950–915,464 nt
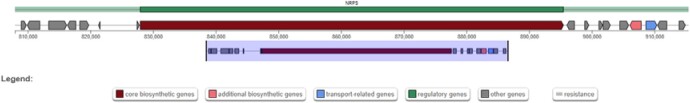	
Predicted sequence	XPXXPXXP?X??XX?XXX??
Trichovirin II 6 b (*Trichoderma viride*)	AcZGALXQXVZGXZPLZZQLeuol

The peptaibol Trichovirin from *T. viride* is cited as an example (Jaworski *et al.* 1999). The letters X and Z stand for isovaline and α-aminoisobutyric acid, respectively. Question marks indicate non-specified residues.

In conclusion, we present the complete genome sequences of the fungi *M.verrucaria*, *M.elegans*, *R.roseolus*, and *S.broomeana.* While our screens of the genomes for genes encoding RiPP precursor proteins of verrucamides, mariannamides, broomeanamides, and borosins did not yield any hits, we discovered 3 candidate NRP biosynthetic gene clusters that may control verrucamide, mariannamide, and broomeanamide biosynthesis, as well as multiple clusters predicted to produce peptaibol-like peptides. These gene clusters will be interesting targets for future research, particularly with regard to the antibacterial properties of verrucamides, mariannamides, and peptaibols (Zou *et al.* 2011; de la Fuente-Núñez *et al.* 2013; Ishiuchi *et al.* 2020).

## Data availability

All relevant data was submitted to ENA with the study accession number PRJEB50709 (secondary accession number ERP135330). The accession numbers of samples, raw reads, and unannotated assemblies are available in Supplementary Table 1. The genome assemblies, predicted gene features and sequences, and antiSMASH annotations are archived on Zenodo with the DOI https://doi.org/10.5281/zenodo.7032226.

Supplemental material is available at *G3* online.

## Supplementary Material

jkac095_Supplementary_Data
