# Correction: Genome sequences of *Rhizopogon roseolus*, *Mariannaea elegans*, *Myrothecium verrucaria*, and *Sphaerostilbella broomeana* and the identification of biosynthetic gene clusters for fungal peptide natural products

**DOI:** 10.1093/g3journal/jkac265

**Published:** 2022-10-18

This is a correction to: Genome sequences of *Rhizopogon roseolus*, *Mariannaea elegans, Myrothecium verrucaria*, and *Sphaerostilbella broomeana* and the identification of biosynthetic gene clusters for fungal peptide natural products, *G3 Genes|Genomes|Genetics*, Volume 12, Issue 7, July 2022, https://doi.org/10.1093/g3journal/jkac095

An emendation is made from the originally published version of this manuscript within section **Data availability**. The final sentence of the first paragraph should read: “The genome assemblies, predicted gene features and sequences, and antiSMASH annotations are archived on Zenodo with the DOI https://doi.org/10.5281/zenodo.7032226.” instead of: “The antiSMASH annotations are available on github under https://microbiologyethz.github.io/vogt_fungal_genomes_2022/.”

This has now been emended within the article.

