# Supplementary material for: Genome sequences of Rhizopogon roseolus, Mariannaea elegans, Myrothecium verrucaria, and Sphaerostilbella broomeana and the identification of biosynthetic gene clusters for fungal peptide natural products
Source: G3 (Bethesda). 2022 Apr 26;12(7):jkac095. doi: 10.1093/g3journal/jkac095 (PMC9258550; doi:10.1093/g3journal/jkac095)
Supplement: jkac095_Supplementary_Data [file jkac095_supplementary_data.docx]

| **Samples** |  |  | **Reads*** |  | **Unannotated assemblies** |
| --- | --- | --- | --- | --- | --- |
| Name | Accession | Biosample | Accession | Experiment | Accession |
| Myrothecium verrucaria  DSM 2087 | ERS10521321 | SAMEA12922176 | ERR8520425  ERR8520426 | ERX8086904  ERX8086903 | ERZ5159763 |
| Mariannaea elegans  NBRC102301 | ERS10521322 | SAMEA12922177 | ERR8520427 | ERX8086902 | ERZ5159777 |
| Rhizopogon roseolus  Mykothek Nr 97.03 | ERS10521323 | SAMEA12922178 | ERR8520428  ERR8520429 | ERX8086901  ERX8086900 | ERZ5159795 |
| Sphaerostilbella broomeana TFC201724 | ERS10521324 | SAMEA12922179 | ERR8520430 | ERX8086899 | ERZ5159804 |

Table S1: The accession numbers of samples, raw reads and unannotated assemblies of all four sequenced fungal genomes (Reads*: two samples were split over multiple flowcells).
